# Characterization of the emerging multidrug-resistant *Salmonella enterica* serovar Indiana strains in China

**DOI:** 10.1080/22221751.2018.1558961

**Published:** 2019-01-16

**Authors:** Jiansen Gong, Ximin Zeng, Ping Zhang, Di Zhang, Chengming Wang, Jun Lin

**Affiliations:** a Poultry Institute, Chinese Academy of Agricultural Sciences, Yangzhou, People’s Republic of China; b Jiangsu Co-Innovation Center for the Prevention and Control of Important Animal Infectious Disease and Zoonose, Yangzhou University, Yangzhou, People’s Republic of China; c Department of Animal Science, The University of Tennessee, Knoxville, TN, USA; d Department of Pathobiology, Auburn University College of Veterinary Medicine, Auburn, AL, USA

**Keywords:** *Salmonella* Indiana, emerging pathogen, multidrug resistance, molecular basis, integron

## Abstract

Emergence of multidrug-resistant (MDR) *Salmonella enterica* serovar Indiana (*S*. Indiana), a dominant *Salmonella* serovar in China, has raised global awareness because the MDR *S*. Indiana also was rapidly emerged in other countries recently. To improve our understanding of underlying MDR mechanism and evolution of this emerging zoonotic pathogen, here we examined the standard ATCC51959 strain together with 19 diverse and representative Chinese *S*. Indiana strains by performing comprehensive microbiological, molecular, and comparative genomics analyses. The findings from S1-PFGE, plasmid origin analysis and Southern blotting suggested the MDR phenotype in the majority of isolates was associated with large integron-carrying plasmids. Interestingly, further in-depth analyses of two recently isolated, plasmid-free MDR *S.* Indiana revealed a long chromosomal class I integron (7.8 kb) that is not linked to the *Salmonella* Genome Island 1 (SGI1), which is rare. This unique chromosomal integron shares extremely high similarity to that identified in a MDR *E. coli* plasmid pLM6771 with respect to both genomic organization and sequence identity. Taken together, both plasmid and chromosomal integron I exist in the examined MDR *S*. Indiana strains. This timely study represents a significant step toward the understanding of molecular basis of the emerging MDR *S.* Indiana.

## Introduction

Antimicrobial resistant bacteria pose a significant threat for livestock production, food safety and public health [1]. Recently, the frequent reports of Gram-negative superbugs, particularly those resistant to extended-spectrum cephalosporins, carbapenem, and colistin, have aroused global concern [2]. *Salmonella* spp. are important foodborne pathogens, to which the elderly, children and immunocompromised people were more susceptible. Antibiotics are still the priority choice for prevention and treatment of salmonellosis because *Salmonella* spp. usually show a lower resistance rate than other Gram-negative bacteria, such as *Escherichia coli* and *Klebsiella* spp [3]. However, the recent emergence of multidrug-resistant (MDR) *Salmonella enterica* serovar Indiana (*S*. Indiana) in China has raised global awareness [4].

*S*. Indiana was first isolated in 1955 from a 9-month-old girl with vomiting, diarrhoea and fever in Indiana, USA. Subsequently, there have been several case reports and outbreaks of *S*. Indiana-causing vomiting, diarrhoea, fever, gastroenteritis, and abortion in humans and animals in North America and Europe, and sporadic infection reports in the other regions (Africa, South America and Oceania) [4]. In China, since the first isolation of *S*. Indiana from a foreign visitor in 1984, there have been only six case reports for *S*. Indiana with a total of 13 isolates being recovered in the subsequent 24 years (1984–2007) [4]. Since 2008, there has been a drastic increase in the number of clinical reports for the infections caused by *S*. Indiana; notably, the increasing prevalence of *S.* Indiana in human, animal, food and environment has made *S.* Indiana become a dominant *Salmonella* serovar in China, replacing the traditionally dominant serovars, such as *S*. Typhimurium, *S*. Derby and *S*. Agona [4]. More importantly, in parallel to the increased prevalence of *S*. Indiana, a trend of emergence of highly MDR *S.* Indiana was also observed [4].

Based on published information, almost all the *S*. Indiana isolates in China were MDR strains [4]. It is important to mention that nearly 90% of the *S*. Indiana isolates were resistant to both ciprofloxacin and ceftriaxone/cefotaxime, which are commonly used to treat severe non-Typhi *Salmonella* infections in adults and children [4]. Moreover, newly identified *S*. Indiana strains also showed resistance to carbapenem, the last-resort antibiotic for the treatment of MDR infections, suggesting that MDR *S*. Indiana has become a serious problem [5]. Alarmingly, the MDR *S*. Indiana was also reported in other countries recently, from Southeast Asia countries to Africa [6–11], suggesting a quick global emergence and expansion of this zoonotic pathogen. Therefore, the MDR *S.* Indiana is posing an increasing threat to animal health and public health globally.

Extensive studies on other *Salmonella* serovars have shown that MDR *Salmonella* strains usually contain unique mobile genetic elements, such as integrons. The integrons were commonly found on conjugative plasmids or in chromosome within the *Salmonella* Genome island 1 (SGI1) [12]. However, surprisingly, the SGI1 was not identified in *S.* Indiana based our previous study [13], which indicates the uniqueness of antibiotic resistance (AR) and evolution of *S.* Indiana when compared to other *Salmonella* serovars. To date, most *S*. Indiana studies only focused on the resistance phenotype and the resistance mechanism with respect to the specific drug [4]. Therefore, systematic and genomics study using diverse strains are highly warranted to reveal underlying MDR mechanisms and likely other unique features of *S*. Indiana [4]. In this study, 19 representative and diverse *S*. Indiana isolates together with the standard control strain ATCC 51959 were subjected to comprehensive microbiological, molecular, and comparative genomics analyses, which not only revealed wide prevalence of class I integron in the MDR isolates but also discovered a unique long chromosomal integron (7.8-kb) that is not associated with SGI1 as observed in other *Salmonella* serovars.

## Results

### Antibiogram of the tested S. Indiana strains

We examined a total of 20 representative *S*. Indiana strains in this study (Table 1). All the *S*. Indiana strains (Table 1), except for ATCC 51959, displayed MDR phenotype with resistance to up to 16 antimicrobials (Table 2). In total, 15 resistance patterns were identified and none of the strains was resistant to imipenem and amoxicillin/clavulanic acid (Table 2). Based on the number and spectrum of tested antimicrobials, the *S.* Indiana strains examined in this study were roughly divided into 4 groups (Table 2). The Group 1 includes ATCC51959 only, which was susceptible to all tested antimicrobials. The Group 2 includes the four early isolates S0802, S1104, S1105, and S1106 (2008-2011), which exhibited variable resistance to 3–8 commonly used antimicrobials, such as ampicillin, streptomycin, tetracycline, and fluoroquinolones (Table 2). Compared to Group 2 strains, the Group 3 showed more consistent resistance to a broader range of antimicrobials with additional new resistance to gentamicin, amikacin and nitrofurantoin (Table 2). However, none of the isolates in Group 3 displayed resistance to the third-generation cephems (cefotaxime, ceftriaxone and ceftazidime) and aztreonam. In contrast, the Group 4 isolates, most of which were isolated in 2014 and 2015, not only exhibited resistance to a broad range of antibiotics as group-3 isolates but also showed resistance to the cephems and aztreonam (Table 2).

**Table 1.** The *S*. Indiana isolates used in this study.

Strain	Time, host, and location of *S*. Indiana collection	Reference
S0802	2008; patients; Shanghai, East China	This study
S1064	2010; broiler chicken; Shandong, East China	[13]
S1104	2011; patients; Shanghai, East China	This study
S1105	2011; aquatic product; Shanghai, East China	This study
S1106	2011; raw chicken; Shanghai, East China	This study
S1204	2012; broiler chicken; Xinxiang, Henan, Central China	[13]
S1318	2013; goose; Danyang, Jiangsu, East China	This study
S1319	2013; broiler chicken; Bazhou, Hebei, North China	[13]
S1402	2014; duck; Jiangyin, Jiangsu, East China	This study
S1407	2014; duck; Wuxi, Jiangsu, East China	This study
S1430	2014; duck; Gaoyou, Jiangsu, East China	This study
S1443	2014; poultry worker; Xuzhou, Jiangsu, East China	[13]
S1445	2014; broiler chicken; Xiaoxian, Anhui, East China	[13]
S1447	2014; pigeon; Taixing, Jiangsu, East China	This study
S1454	2014; broiler chicken; Zhaoxian, Hebei, North China	[13]
S1459	2014; broiler chicken; Quyang, Hebei, North China	[13]
S1467	2014; broiler chicken; Xuzhou, Jiangsu, East China	This study
S1501	2015; layer chicken; Fengxian, Jiangsu, East China	This study
S1515	2015; duck; Peixian, Jiangsu, East China	This study

**Table 2.** Antibiotic resistance phenotype of the tested *S.* Indiana strains.

Strain (Isolation year)	Group	Antibiotics																		
		AMP	AMC	CTX	CRO	CAZ	ATM	IPM	STR	GEN	KAN	AMK	TET	NAL	CIP	SUL	TMP	SXT	CHL	NIT
ATCC51959	1																			
S0802 (2008)	2	R							R		R		R	R	R	R			R	
S1104 (2011)									R		R			R	R	R	R	R	R	
S1105 (2011)													R	R	R					
S1106 (2011)		R							R				R			R	R	R	R	
S1064 (2010)	3	R							R	R	R		R	R	R	R	R	R	R	R
S1319 (2013)		R							R	R	R	R	R	R	R	R	R	R	R	R
S1402 (2014)		R							R		R		R	R	R	R	R	R	R	
S1430 (2014)		R							R	R	R	R	R			R	R	R		R
S1204 (2012)	4	R		R	R		R		R	R	R	R	R	R	R	R	R	R	R	R
S1318 (2013)		R		R	R	R	R		R	R	R			R	R	R	R	R	R	R
S1407 (2014)		R		R	R				R	R	R		R	R	R	R	R	R	R	
S1443 (2014)		R		R	R	R	R		R	R	R		R	R	R	R	R	R	R	R
S1445 (2014)		R		R	R	R	R		R	R	R		R	R	R	R	R	R	R	R
S1447 (2014)		R		R	R	R	R		R	R	R		R	R	R	R	R	R	R	R
S1454 (2014)		R		R	R		R		R	R	R	R	R	R	R	R	R	R	R	R
S1459 (2014)		R		R	R	R	R		R	R	R		R	R	R	R	R	R	R	R
S1467 (2014)		R		R	R	R	R		R	R	R	R	R	R	R	R	R	R	R	
S1501 (2015)		R		R	R	R	R		R	R	R	R	R	R	R	R	R	R	R	
S1515 (2015)		R		R	R		R		R	R	R		R	R	R	R	R	R	R	

Notes: AMP: ampicillin; AMC: amoxicillin/clavulanic acid; CTX: cefotaxime; CRO: ceftriaxone; CAZ: ceftazidime; ATM: aztreonam; IPM: imipenem; STR: streptomycin; GEN: gentamicin; KAN: kanamycin; AMK: amikacin; TET: tetracycline; NAL: nalidixic acid; CIP: ciprofloxacin; SUL: sulfafurazole; TMP: trimethoprim; SXT: trimethoprim/sulfamethoxazole; CHL: chloramphenicol; NIT: nitrofurantoin; R: resistant.

### Genome mining of MDR determinants

The sequencing coverage and depth were summarized in Table S1 (Supplementary Information). The d*e novo* assembled contigs of the 20 *S.* Indiana strains were initially aligned against the recently published *S.* Indiana C629 genome [14]. This initial comparative genome analysis identified limited MDR genes in our *S.* Indiana strains that displays homology to those present in the C629 plasmid pRCW1 conferring resistance to aminoglycoside, beta-lactam, fluoroquinolone, phenicol, sulphonamide, and trimethoprim [14].

Further comprehensive gene mining using ResFinder [15] for the assembled contigs from each *S.* Indiana isolates revealed a total of 45 different AR genes in these diverse *S.* Indiana strains, which may confer resistance to 10 classes of antimicrobials (Table S2). The AR gene profiles of those *S.* Indiana strains (Table S2) are highly consistent with their resistance phenotypes (Table 2). For example, ATCC 51959, which is sensitive to most antibiotics, carries the least extent of AR genes, while S1467 and S1501, which are resistant to the most antibiotics (Table 2), carry the broadest spectrum of AR genes (Table S2, Supplementary Information). Interestingly, strain S1318 is susceptible to tetracycline although it carries intact *tetA* gene and there is no mutation detected in *tetA* and adjacent regulator *tetR*, suggesting potential regulatory suppression of *tetA* in this strain.

### Identification and characterization of class I integron in S. Indiana

We observed that a panel of AR genes is clustered in the sequenced *S.* Indiana genomes. However, the AR gene-bearing plasmid pRCW1 from *S.* Indiana C629 [14] does not have typical integron due to the lack of conserved signature regions (Data not shown). Thus, simple comparison of the identified AR genes from the 19 *S.* Indiana strains to the C629 genome cannot help us to link the clustered MDR genes to potential mobile genetic elements in *S.* Indiana, such as integron.

**Figure 1.**
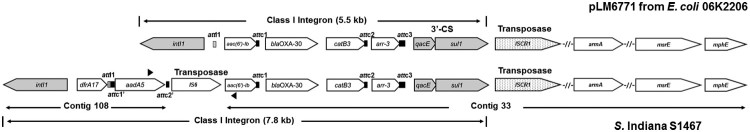
Comparison of class I integron in the pLM6771 and the *S*. Indiana strain S1467. The complete class I integron in *S*. Indiana S1467 was revealed by filling the gap between the Contig 108 and Contig 33 using primer pair of Ct108 and Ct33 (Table S3; depicted by solid triangles). The grey arrows indicate the class I integron-associated *intI1* gene and the typical 3’-conserved segment (3’-CS) containing *qacE* and *sul1* genes. The grey and solid rectangles represent the critical signature sequences of the class I integron (*attI* and *attC* sites). The dotted arrow represents the unique transposase gene *ISCR1* immediately downstream of the class I integron. The open arrows denote various AR genes (detailed annotations in Table 3).

Interestingly, further database searches revealed that a panel of unique AR gene cassettes in our *S.* Indiana isolates displayed high level similarity to those in the large plasmid pLM6771 from *E. coli* 06K2206 (89,090 bp; GenBank accession number: KX009507). Notably, a complete class I integron in pLM6771 was identified using IntegronFinder [16], which is characterized by the presence of one *int*I1, one *att*I, three *att*C sites, and the conserved segment of the integron at 3’ end (3'-CS) comprised of the *qacE* and *sul1* genes (Figure 1). Other AR genes were also identified within this integron in the pLM6771, which include aminoglycoside N(6)-acetyltransferase gene *aac(6’)-Ib*, beta-lactamase gene *bla*OXA-30, chloramphenicol acetyltransferase gene *catB3*, rifampin ADP-ribosylating transferase gene *arr-3* (Figure 1). In addition, immediately following the 3'-CS, a single copy of insertion sequence common region 1 (ISCR1) gene was identified in pLM6771 (Figure 1), which encodes a transposase frequently associated with a specific type of class I integron [17]. Finally, additional AR genes adjacent to this integron were also identified in pLM6771, such as those encoding ribosomal RNA methyltransferase ArmA, macrolide efflux protein MsrE, macrolide 2'-phosphotransferase MphE, beta-lactamase IRT-4, beta-lactamase CTX-M-3; the *armA*, *msrE*, and *mphE* genes are depicted in Figure 1.

**Table 3.** Distribution of the major *E. coli* pLM6771-associated AR genes and mobile elements in different *S.* Indiana strains.

AR Gene^a^	Annotation/Function	ATCC 51959	S0802	S1104	S1105	S1106	S1064	S1319	S1402	S1430	S1204	S1318	S1407	S1443	S1445	S1447	S1454	S1459	S1467	S1501	S1515
*bla*_IRT-4_	Beta-lactamase IRT-4	N/A^c^	Y	N/A	Pa	N/A	Pa	Y	Y	Y	Y	Pa	N/A	Y	N/A	Y	Y	N/A	N/A	N/A	Pa
*IntI1*	Integrase IntI1	Y	N/A	Y	Y	Y	Y	Y	Y	y	Y	Y	Y	Y	Y	Y	Pa	y	Y	Y	Y
*aac(6’)-Ib*	Aminoglycoside N(6’)-acetyltransferase	N/A	Pa	N/A	Pa	Y	Y	Y	Y	Y	Pa	Y	Y	Y	Y	Y	Y	Y	Y	Y	Y
*bla*_OXA-30_	Beta-lactamase OXA-30	Pa^d^	Pa	N/A	Pa	Y	Y	Y	Y	Y	Y	Y	Y	Y	Y	Y	Y	Y	Y	Y	Y
*catB3*	Chloramphenicol acetyltransferase	N/A	N/A	N/A	Pa	Y	Y	Y	Y	Y	Y	Y	Y	Y	Y	Y	Y	Y	Y	Y	Y
*arr-3*	Rifampin ADP-ribosylating transferase	Pa	N/A	N/A	Pa	Pa	Y	Y	Y	Y	Y	Y	Y	Y	Y	Y	Y	Y	Y	Y	Y
*qacE*^b^	Quaternary ammonium efflux transporter	N	N/A	Pa	Pa	Y	Y	Y	Y	N	Y	Pa	Y	Pa	Pa	Pa	Y	Y	Y	Y	Y
*sul1*	Dihydropteroate synthase Sul1	Y^e^	N/A	Y	Pa	Y	Y	Y	Y	N	Y	N	Y	N	N	N	Y	y^g^	Y	Y	Y
*iscr1*	Transposase ISCR1	N/A	N/A	N/A	Pa	Pa	Pa	Y	Pa	Pa	V	V	Pa	N	V	Y	Y	Y	Y	Y	V
*armA*	Aminoglycoside resistance methylase	N/A	N/A	N/A	N/A	N/A	N/A	N/A	N/A	N/A	N/A	N/A	N/A	N/A	N/A	N/A	Y	y	Y	Y	N/A
*msrE*	Macrolide efflux protein	N/A	N/A	N/A	N/A	N/A	N/A	N/A	N/A	N/A	N/A	N/A	N/A	N/A	N/A	N/A	N/A	y	Y	Y	N/A
*mphE*	Macrolide 2'-phosphotransferase	N/A	N/A	N/A	N/A	N/A	N/A	N/A	N/A	N/A	N/A	N/A	N/A	N/A	N/A	N/A	N/A	Y	Y	Y	N/A
*bla*_CTX-M-3_	CTX-M-3 beta-lactamase	Pa	N/A	N/A	N/A	N/A	N/A	N/A	V^f^	V	Pa	Y	V	V	V	V	V	y	Y	Y	V

^a^The AR gene from the plasmid pLM6771 in *E. coli* 06K2206.

^b^*qacE* is present but was not annotated in published pLM6771.

^c^N/A, not available. It means the corresponding AR gene sequence was not identified in comparison using Mauve.

^d^Pa detonates “partial”; only partial AR gene sequence was aligned to pLM6771. Missing portion of AR gene sequences might be in other small contigs, which failed to align to pLM6771.

^e^Y, the full length open reading frame (ORF) of corresponding AR gene sequence was identified and well aligned to pLM6771.

^f^V denotes “variable”; the potential AR gene in *S.* Indiana strains displayed significant sequence variation to the corresponding AR gene in pLM6771.

^g^y, near full length of ORF of corresponding AR gene sequence was identified through the comparison using Mauve.

Strikingly, similar genomic organization of the same AR gene cluster in the *E. coli* plasmid pLM6771 was directly observed in some assembled *S.* Indiana genomes, such as *S.* Indiana strain S1467 and S1501. As shown in Figure 1, the Contig 108 for S1467 possesses the same signature sequences (*int*I1, *att*I, and *att*C sites) plus additional *dfrA17* and *aadA5* at the other end; the Contig 33 shares extremely high similarity to the class I integron and its downstream genes carried in pLM6771 with respect to both genomic organization and sequence identity. Using primer pair of Ct108 and Ct33 (Table S3), the gap between Contig 108 and Contig 33 was filled, revealing another transposase gene *IS6* (Figure 1). The IS6 is also located in a short Contig 98 that seems a repeated region. The complete long class I integron identified in S1467 strain (7.8 kb) is depicted in Figure 1.

In other *S.* Indiana strains, despite distribution of various AR genes in different contigs, the similar class I integron components and genomic organization of AR genes as shown in Figure 1 were also observed, strongly suggesting that these *S.* Indiana strains also have similar class I integron. According to our exhaustive genome sequence analysis, distribution of pLM6771-associated AR genes as well as mobile elements in different *S.* Indiana strains is summarized in Table 3. Notably, the core four AR genes within the identified integron, *aac(6’)-Ib*/*bla*OXA-30/*catB3*/*arr-3* (Figure 1), have been discovered in majority of the MDR *S.* Indiana strains (Table 3).

### Plasmid analysis: prevalence, origins and location of the class I integron

**Figure 2.**
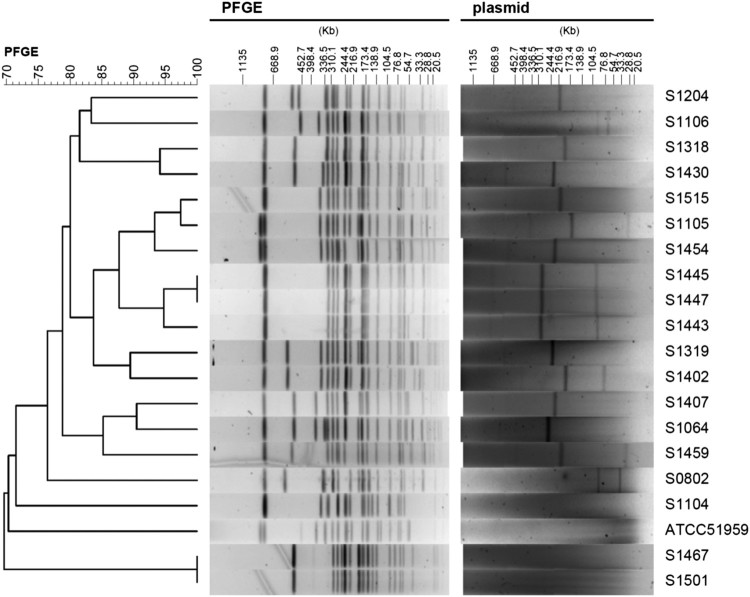
PFGE and S1-PFGE analyses of *S*. Indiana strains. The left panel is the phylogenetic tree of *S*. Indiana strains. The middle and right panel is the corresponding PFGE and S1-PFGE result, respectively.

**Figure 3.**
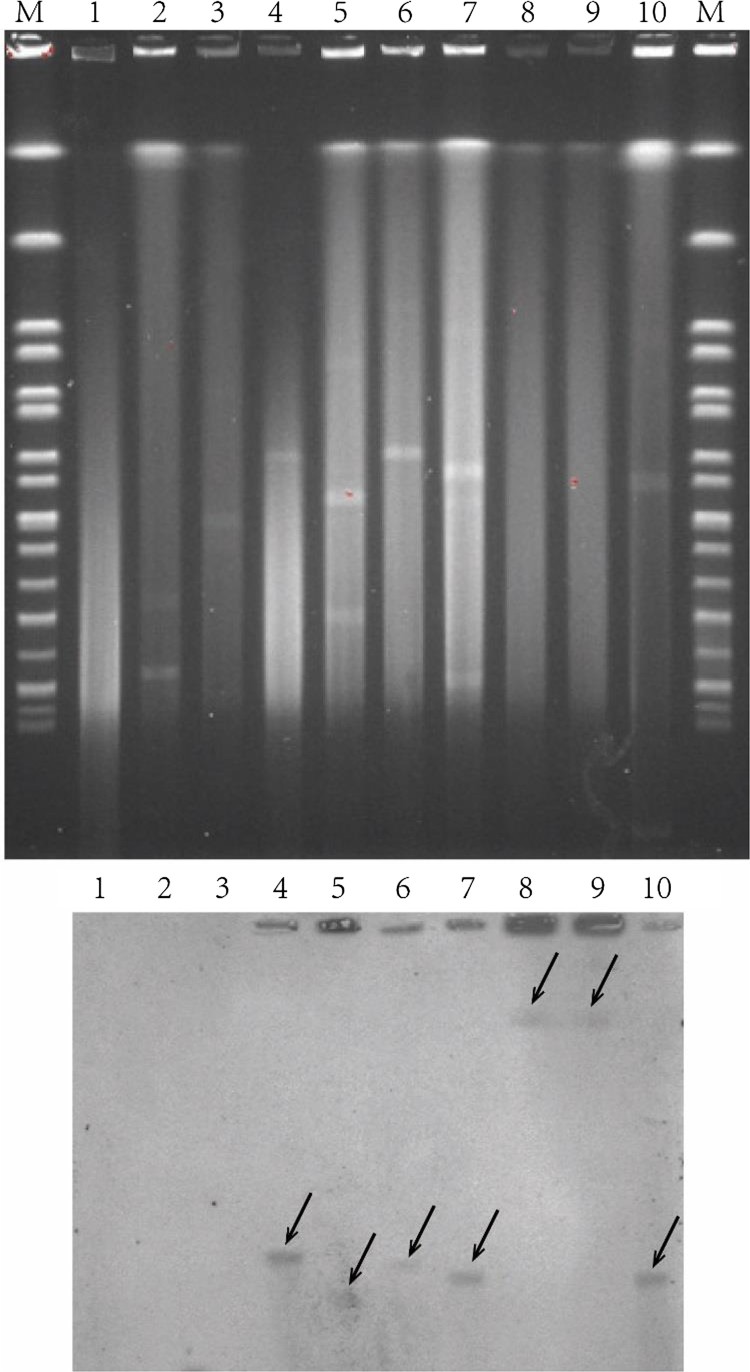
Location of class I integron in *S*. Indiana strains. S1-PFGE was performed for selected *S.* Indiana isolates (upper panel). Separated DNA was subsequently transferred into nylon membrane and subjected to Southern blot hybridization against the class I integron by targeting *qac*E (bottom panel). Lane M, marker H9812; lane 1, ATCC51959; lane 2, S0802; lane 3, S1105; lane 4, S1319; lane 5, S1402; lane 6, S1407; lane 7, S1459; lane 8, S1467; lane 9, S1501; lane 10, S1515.

S1-PFGE showed that plasmids were observed in 16 out of 20 *S.* Indiana strains, and most of them harboured large plasmids (>200 kb) (Figure 2). It seems that the strains ATCC51959, S1104, S1467 and S1501 do not carry any plasmids (Figure 2). However, S1-PFGE approach might miss small DNA fragments (including small plasmids). To further determine if these four strains are indeed plasmid-free, we used two complementary approaches: bioinformatics analysis of plasmid origin and direct plasmid extraction. The assembled contigs from all 20 strains were analysed for plasmid origins using PlasmidFinder [18]. As shown in Table 4, the strain S1467 and S1501 did not carry any known plasmid origins, suggesting they are free of plasmids. Furthermore, plasmid extraction using Plasmid Miniprep Kit (Qiagen) did not identify any plasmids in S1467 and S1501 (Figure S1), indicating lack of small plasmids (<20 kb) in these two *S.* Indiana strains. Therefore, given that both S1467 and S1501 possess most of AR genes (Table 3) and the complete long class I integron as shown in Figure 1, the MDR phenotype in S1467 and S1501 should be conferred by the integron located in chromosome. This conclusion is further confirmed by S1-PFGE together with Southern blot analysis. As shown in Figure 3, Southern blot hybridization using specific probe (targeting *qac*E) demonstrated that the plasmid-mediated class I integrons were indeed located in the large plasmids of representative *S.* Indiana isolates (S1319, S1402, S1407, S1459 and S1515), while the integrons in the plasmid-free isolates (S1467 and S1501) were located in chromosome (Figure 3).

**Table 4.** Analyses of the plasmids in different *S.* Indiana strains.

Plasmid	ATCC51959	S0802	S1064	S1104	S1105	S1106	S1204	S1318	S1319	S1402	S1407	S1430	S1443	S1445	S1447	S1454	S1459	S1467	S1501	S1515
*Plasmid Origin*	** **	** **	** **	** **	** **	** **	** **	** **	** **	** **	** **	** **	** **	** **	** **	** **	** **	** **	** **	** **
Col156	** **	** **	** **	** **	** **	** **	** **	**+**	**+**	**+**	**+**	** **	** **	** **	** **	** **	** **	** **	** **	** **
Col8282	** **	** **	** **	** **	** **	** **	** **	** **	** **	** **	** **	** **	** **	** **	** **	**+**	** **	** **	** **	** **
ColRNAI	** **	** **	** **	** **	** **	** **	** **	** **	** **	** **	** **	** **	** **	** **	**+**	** **	** **	** **	** **	** **
IncFIB (K)	** **	** **	** **	** **	**+**	**+**	** **	** **	** **	** **	** **	** **	** **	** **	** **	** **	** **	** **	** **	** **
IncHI2	**+**	** **	**+**	**+**	**+**	**+**	**+**	**+**	**+**	**+**	**+**	**+**	**+**	**+**	**+**	**+**	**+**	** **	** **	**+**
IncHI2A	** **	** **	**+**	**+**	**+**	**+**	** **	**+**	**+**	**+**	**+**	**+**	**+**	**+**	**+**	**+**	**+**	** **	** **	**+**
IncN	**+**	** **	**+**	**+**	** **	** **	** **	**+**	**+**	**+**	** **	**+**	** **	** **	** **	** **	** **	** **	** **	** **
IncQ1	** **	**+**	** **	** **	** **	** **	**+**	**+**	**+**	**+**	** **	**+**	**+**	** **	**+**	**+**	** **	** **	** **	** **
IncX1	**+**	** **	** **	**+**	**+**	** **	** **	**+**	** **	** **	** **	** **	**+**	** **	**+**	**+**	**+**	** **	** **	** **
p0111	** **	**+**	** **	** **	** **	** **	** **	** **	** **	**+**	**+**	** **	**+**	**+**	**+**	** **	** **	** **	** **	** **
*Plasmid #*	*** ***	*** ***	*** ***	*** ***	*** ***	*** ***	*** ***	*** ***	*** ***	*** ***	*** ***	*** ***	*** ***	*** ***	*** ***	*** ***	*** ***	*** ***	*** ***	*** ***
Sequencing^a^	3	2	3	4	4	3	2	6	5	6	4	4	5	3	6	5	3	0	0	2
S1-PFGE^b^	0	2 (41,89)	1 (261)	0	2 (31,168)	2 (68,93)	1 (215)	1 (194)	1 (243)	2 (76,190)	1 (235)	1 (240)	2 (92,296)	2 (96,290)	2 (95,290)	1 (235)	2 (32,210)	0	0	1 (213)

^a^The total number of plasmid origins based on the PlasmidFinder analysis of the assembled genome sequences.

^b^The total plasmid number based on S1-PFGE analysis. The number in parentheses indicates estimated kb size of the identified plasmid.

### Lack of SGI1 in the S. Indiana strains

It has been widely reported that chromosomal integron was located in SGI1 in *Salmonella* [12]. However, based on genome sequence analysis in this study, SGI1 was not observed in any of the examined *S.* Indiana strains. To verify this finding, PCR was performed using a panel of SGI1-specific primers (Table S3, Supplementary Information). Consistently, SGI1 variants were not detected in all the *S.* Indiana strains including S1467 and S1501 (Figure S2). Therefore, the chromosomal class I integrons in S1467 and S1501 are not associated with SGI1, which is rare in *Salmonella*. The finding for the lack of SGI1 in *S.* Indiana in this study, which is supported by whole genome and PCR analyses, also confirmed our previous preliminary observations [13].

### Phylogenetic analysis of S. Indiana strains

As shown in Table S4 (Supplementary Information), all *S.* Indiana strains possess same alleles of the seven housekeeping genes (*Salmonella* 7-gene dataset from Enterobase), making all the tested *S.* Indiana strains fall in the same ST17 type based on the MLST analysis. Although MLST typing indicated the *S.* Indiana strains have a very close phylogenetic relationship, PFGE analysis of the 20 strains (Figure 2) revealed several different patterns. However, some strains, such as S1443 (human strain), S1445 (chicken strain), and S1447 (pigeon strain) which were isolated from different regions, displayed consistent antibiogram, AR gene profile as well as similar PFGE pattern, indicating a potential close or clonal relationship of these isolates. Inconsistency between the phenotype and the genotype was also observed in some strains. For instance, although S1515 and S1105 displayed similar PFGE pattern (Figure 2) and AR gene profile (Table 3), unlike S1515, S1105 is a “silent” strain displaying susceptibilities to a panel of antibiotics (Table 2), which is likely attributed to an unidentified regulatory mechanism for AR genes in S1105.

### Whole genome sequencing typing

**Figure 4.**
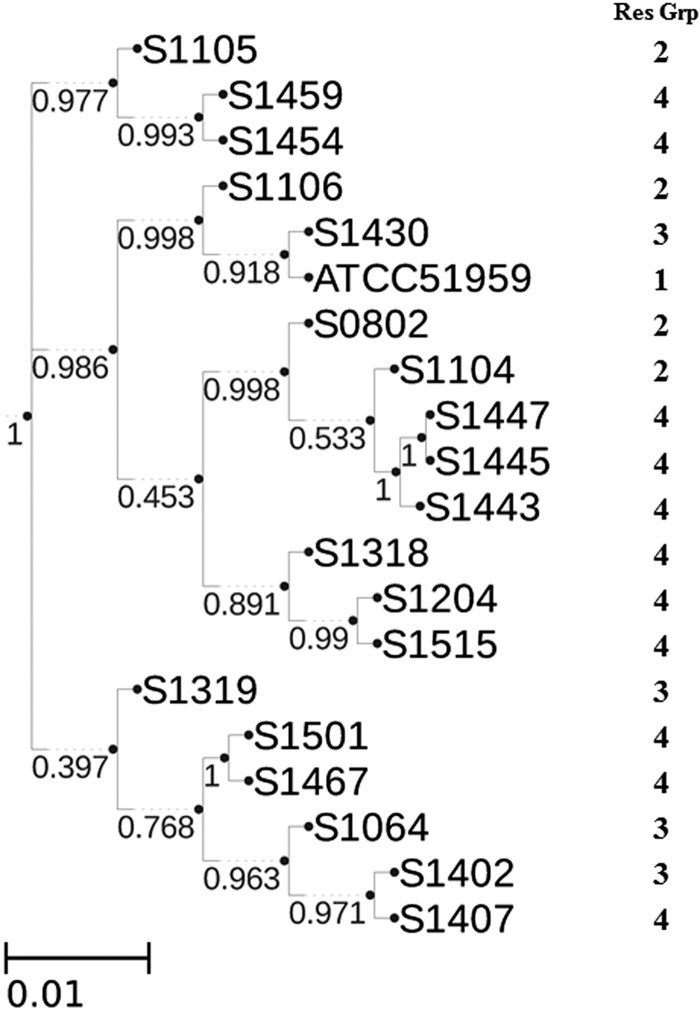
Whole genome SNP tree analysis of *S*. Indiana strains. Assembled contigs from 20 *S*. Indiana strains were analysed by PathoBacTyper. The maximum-likelihood phylogenetic tree was constructed with confidence values labelled on the branches. The length of scale bar indicates 1 nucleotide substitution per 100 sites.

Since the genome-scale genetic information is available, whole genome sequencing typing were performed in the PathoBacTyper web server. The whole genome SNP (wgSNP) tree was shown in Figure 4. The wgSNP tree consists of 3 different clusters. However, the clustering of strains in the wgSNP tree does not seem strictly consistent with the clustering based on resistance phenotype. For example, strain S1105, which is very sensitive to most antibiotics (Table 2), is clustered with S1459 and S1454 in wgSNP tree, which displayed MDR phenotype (Table 2). However, as observed in PFGE analysis, some strains with similar resistant phenotype were placed in proximity in wgSNP tree, such as S1443/S1445/S1447 and S1467/S1501.

## Discussion

Currently, there are over 2600 recognized *Salmonella* serovars. Extensive attentions were placed on those widely prevalent serovars, such as *S*. Enteritidis and *S*. Typhimurium, while most *Salmonella* serovars were considered uncommon serovars. However, some uncommon serovars can arise as prevalent serovars in specific geographic locations [19]. Particularly, recent studies showed that the *S*. Indiana has evolved from an infrequently reported serovar to one of the most common serovar in China [4]. In parallel to the increased prevalence of *S*. Indiana, the rapid rise of MDR in *S.* Indiana also raised serious concerns [4]. Currently, there is a trend of worldwide emergence of the MDR *S*. Indiana [6–11], highlighting the needs of in-depth characterization of the MDR *S.* Indiana. In this timely study, to explore molecular mechanisms of MDR *S*. Indiana, representative Chinese *S*. Indiana strains were selected for comparative phenotypic and genomic characterizations.

AR gene clusters are of particular interests since they confer resistance to several different antimicrobials or classes of antimicrobials. In this study, we found a unique AR gene cluster (or integron) in diverse Chinese *S.* Indiana strains; this integron is strikingly similar to the class I integron carried in the plasmid pLM6771 from *E. coli* 06K2206. According to the submitted information of the pLM6771 genome, the host of this plasmid is a MDR *E. coli* strain from South Korea. We inferred that the integron observed in this *E. coli* plasmid is closely related to the integron identified in recent Chinese MDR *S*. Indiana isolates. However, at this stage, we do not have sufficient epidemiological and genomic evidence demonstrating if this integron was transmitted from *S.* Indiana to *E. coli* or from *E. coli* to *S*. Indiana*.* Given that the integron was shared by different bacterial species from different countries, this unique integron may contribute significantly to the emergence and dissemination of MDR globally in *Enterobacteriaceae,* such as *E. coli* and *Salmonella*. This speculation needs to be examined in large-scale studies through international collaboration in the future.

Integrons, enriched with AR genes, can be efficiently disseminated among bacteria via plasmids, particularly with the aid from other mobile elements such as transposon. Previous studies have proposed that MDR phenotype of *S*. Indiana was likely attributed to the presence of resistant plasmids, including those belonging to IncHI2-type, IncN-type, IncA/C-type, IncP-type and IncFIB-type [13,20,21]. Recently, we observed that a single MDR *S*. Indiana isolate usually carried one or two plasmids [22] and even 4 plasmids with the presence of resistance genes on 3 plasmids [5]. In this study, S1-PFGE has confirmed this multi-plasmid nature in at least 8 strains (Figure 2 and Table 4). The Southern blot hybridization demonstrated that the class I integron is located in the large plasmids of all representative plasmid-containing MDR *S.* Indiana isolates (Figure 3), suggesting the class I integron is effectively disseminated via plasmid. However, given the presence of the similar integron in diverse plasmids, at this stage, it is still unknown which factors (e.g. transposase, environmental signals) play a critical role in the hopping of the class I integron among different plasmids.

It is of particular interests that two MDR *S*. Indiana strains (S1467 and S1501) are free of plasmids. Based on plasmid origin analysis and S1-PFGE together with Southern blot hybridization in this study, we obtained compelling evidence indicating the MDR phenotype of *S*. Indiana strains S1467 and S1501 were conferred by a large chromosomal class I integron. Notably, we also observed that the *E. coli* pLM6771 belongs to IncL/M group but none of our *S.* Indiana strains carry either IncL/M or IncA/C plasmid origins (Table 4); this finding together with the high similarity of the class I integron in pLM6771 plasmid of *E. coli* strain to that in the chromosome of *S.* Indiana S1467 (Figure 1) strongly suggest that the AR gene cluster-containing class I integron can actively hop among plasmids or between plasmid and chromosomes with aid of certain mobile elements, such as the integrase and transposase ISCR1 [17] as shown in Figure 1. Interestingly, we also demonstrated that the chromosomal class I integron present in these two very recent isolates (S1467 and S1501) was not linked to SGI1, which was rare in *Salmonella* spp. Since such unique chromosomal integron was not observed in the early MDR *S*. Indiana, does emergence of this chromosomal integron represent a trend of MDR *S*. Indiana in the future? This question is highly warranted to be addressed in future large epidemiological investigations, particularly in view of the worldwide spread of SGI1 after the identification of MDR *S*. Typhimurium DT104 [23].

MLST analysis showed that all the 20 *S*. Indiana strains belonged to the same MLST type (ST17), which is in agreement with other studies [13,22,24]. According to *Salmonella* MLST database, *S*. Indiana isolates in the areas other than China (including France, Denmark, UK and Scotland) also belong to ST17, indicating that *S*. Indiana exhibited the least sequence types among *Salmonella* serovars. Despite wide prevalence of *S*. Indiana in various sources, clearly, the MLST type of *S*. Indiana is significantly less than those of other prevalent serovars, such as *S*. Enteritidis and *S*. Typhimurium. These MLST typing studies all showed that *S*. Indiana isolates have a very close phylogenetic relationship. Despite many common themes among various *Salmonella* serovars, presence of a single MLST type for the emerging MDR *S.* Indiana suggests this specific *Salmonella* serovar may have acquired unique features to outcompete other serovars during evolution.

To effectively distinguish *S*. Indiana strains from different sources, the PFGE and whole genome sequencing typing were performed as well for the diverse *S*. Indiana strains examined in this study. The PFGE result showed that all the 20 *S*. Indiana strains shared >70% similarity in the PFGE patterns, also the close relationship of the *S*. Indiana strains. In other reports, *S*. Indiana isolates also showed high PFGE similarity (50–80%) without predominant PFGE type identified despite diverse phenotypes, which was different from what has been observed in other prevalent serovars such as *S*. Typhimurium and *S*. Enteritis [20,22,25,26]. Unlike MLST and PFGE, as expected, whole genome sequencing typing provide unprecedented discriminating power to differentiate highly close lineages. By sequencing the entire genome, most genetic information, such as bacterial classification, AR genes, virulent genes, and SNPs, could be revealed. Besides that, the possible horizontal gene transfer method (via mobile genetic elements) could be analysed. However, the nature of unfinished genomes from high-throughput sequencing still impeded us to get clues for some intriguing findings. For example, although some strains, such as S1515 and S1105, exhibited similar PFGE pattern (Figure 2) and AR gene profile (Table 3), they displayed dramatically different AR phenotype (Table 2). This interesting finding is likely due to unknown mechanisms of AR gene regulation [27–29]. To test this, obtaining finished genomes of these two isolates are highly warranted in the future.

Although whole genome analysis of the diverse MDR *S.* Indiana strains in this study has led to novel discovery on molecular basis of the rapidly emerging multidrug resistance in *S.* Indiana, extensive genome analysis did not help us identify meaningful clues for the evolution of MDR *S.* Indiana. We did not find unique gene or genomic island that may enable *S.* Indiana to successfully outcompete the traditionally dominant *Salmonella* serovars (e.g. *S*. Typhimurium and *S*. Derby) although *S.* Indiana has become a dominant *Salmonella* serovar in China in the past decade. The MDR S. Indiana also seems to emerge and expand rapidly to other countries based on recent reports [6–11]. Using a univariate and multivariate analysis, recently Collignon et al. [30] proposed that the spread of resistant strains and resistance genes seems to be the dominant contributing factor for the threatening antimicrobial resistance. Therefore, regarding the intriguing *S.* Indiana, it is likely the MDR *S.* Indiana acquires significantly enhanced *in vivo* adaptation during evolution when compared to the other existing MDR *Salmonella* serovars. To test this, comprehensive assessments of *S.* Indiana colonization in the host (e.g. chicken) using representative *S.* Indiana strains and other *Salmonella* serovar strains are critically needed in the future. Such animal studies in conjunction with in-depth studies using various omics approaches may greatly improve our understanding of *S.* Indiana evolution and help us develop effective strategies to control the global emergence of MDR *S.* Indiana.

## Materials and methods

### S. Indiana strains

A total of 20 representative *S*. Indiana strains were selected and examined in this study, which include the standard strain ATCC51959 and 19 Chinese isolates (Table 1). For these Chinese *S*. Indiana isolates, 7 isolates (6 from broilers and 1 from poultry worker) was selected from our previous *S*. Indiana epidemiological study [13], and the remaining 12 isolates was recovered from other sources. To ensure diversity and representativeness, the selection of the 19 Chinese *S.* Indiana isolates is primarily based on differences in host, isolation year, and geography. Specifically, these diverse *S*. Indiana strains isolated from multiple sources (Table 1), such as human (diarrhoea patients, poultry worker), food (aquatic products, raw chicken) and animal (chicken, duck, goose, pigeon). These *S*. Indiana isolates are also geographically diverse, which were collected at 16 sites in six provinces in China between 2008 and 2015 (Table 1).

### Antimicrobial susceptibility testing

All the *S*. Indiana strains were tested for antimicrobial susceptibility using the disk diffusion method according to the Clinical and Laboratory Standards Institute guidelines (CLSI, 2013). The *S*. Indiana strains were tested against 20 antimicrobial agents (OXOID): ampicillin (10 μg), amoxicillin/clavulanic acid (20/10 μg), cefotaxime (30 μg), ceftriaxone (30 μg), ceftazidime (30 μg), aztreonam (30 μg), imipenem (10 μg), streptomycin (10 μg), gentamicin (10 μg), kanamycin (30 μg), amikacin (30 μg), tetracycline (30 μg), nalidixic acid (30 μg), ciprofloxacin (5 μg), sulfafurazole (300 μg), trimethoprim (5 μg), sulfamethoxazole/trimethoprim (23.75/1.25 μg), chloramphenicol (30 μg), and nitrofurantoin (300 μg). The *E. coli* ATCC 25922 was used as a quality control strain.

### Pulsed-field gel electrophoresis (PFGE) and Southern blotting

PFGE of the macrorestriction fragment patterns of genomic DNA using *Xba*I enzyme was performed by following the Centers for Disease Control and Prevention (CDC) standardized PulseNet protocol for *Salmonella* [31]. S1-PFGE was performed to determine the plasmid profile as described previously [32]. The PFGE profiles of these isolates were clustered by the unweighted-pair group method using average linkages (UPGMA). The isolates whose PFGE patterns displayed a similarity coefficient of >90% were considered as a PFGE cluster having closely related (clonal) genotypes [33]. The location of class I integron was indicated by Southern blot hybridization using a digoxigenin-labelled *qacE* probe (generated with primer *qacE*-F/qacE-R by using *S*. Indiana S1467 genomic DNA as template) according to the manufacturer’s instructions for the DIG-High Prime DNA Labelling and Detection Starter Kit II (Roche Diagnostics, Mannheim, Germany). Ten *S*. Indiana strains were chosen for Southern blot hybridization, including ATCC51959 (lack of class I integron), S0802 and S1105 (contained incomplete class I integrons), S1319, S1402, S1407, S1459 and S1515 (class I integrons located in plasmids), S1467 and S1501 (class I integrons located in chromosome).

### Genome sequencing and comparative genomics analysis

Genomic DNA was extracted using DNeasy Blood & Tissue Kit (Qiagen, 69506). The genomic DNA from all the 20 *S.* Indiana strains were subjected to whole genome sequencing using HiSeq 2500 Sequencing platform at Shanghai Biotechnology Corporation (Shanghai, China), where genomic DNAs were end repaired, ligated to specific adaptors and subject to paired-end sequencing. After filtering raw reads, the clean reads were *de novo* assembled into contigs using the CLC Genomics Workbench (Genomics Hub, The University of Tennessee, USA). The contigs were aligned against the reference genome of *S.* Indiana C629 [14] and *E. coli* 06K2206 plasmid LM6771 (GenBank No: KX009507) by using Mauve (v 2.3.0) [34,35]. *S.* Indiana C629 was chosen as a template because it was the only *S.* Indiana strain with full genome sequence available when this project was performed. The assembled contigs from each *S.* Indiana strains were also uploaded to the Center for Genomic Epidemiology (http://www.genomicepidemiology.org/) for searching acquired AR genes [15] and plasmid origins [18].

### Polymerase chain reaction (PCR)

PCR was performed to fill the gap of assembled integron region and to verify the presence of SGI1 variants in *S.* Indiana. PCR primers were described in Table S3, in which the primers of U7-L12/LJ-R1, 104-RJ/C9-L2 and U7-L12/104-D target the SGI1 left junction, right junction and across junction, respectively [36]. The PCR reaction procedure consisted of an initial denaturation step of heating at 95°C for 5 min, 30 cycles of 94°C for 1 min, 56°C for 1 min, and 72°C for 1 min. Then there was a final extension step of 72°C for 10 min. The *S*. Albany strain B0532 (GenBank No: KU288619) and *S*. Enteritidis standard strain CMCC50041 (GenBank No: CP013097) were used as SGI1 positive control and negative control, respectively.

### Multilocus sequence typing (MLST)

Seven conserved house-keeping genes (*aroC*, dnaN, *hemD*, *hisD*, *purE*, *sucA*, and *thrA*) were extracted from assembled contigs, and compared with the *Salmonella* MLST database website (http://mlst.warwick.ac.uk/mlst/dbs/Senterica) to determine the sequence types.

### Whole genome sequencing typing

The whole genome sequencing typing was performed using whole-genome-scale single nucleotide polymorphism (wgSNP) method. The assembled contigs and the reference genome C629 [14] were uploaded to the PathoBacTyper server (http://halst.nhri.org.tw/PathoBacTyper/) for performing wgSNP-based genome typing [37].

### Nucleotide sequence accession number

The raw sequencing reads of the 20 *S.* Indiana strains were deposited into GenBank under BioProject PRJNA420683.

Supplementary information accompanies the manuscript on the Emerging Microbes & Infections website http://www.nature.com/emi.
